# Intertester and intratester reliability of movement control tests on the hip for patients with hip osteoarthritis

**DOI:** 10.1186/s12891-017-1388-5

**Published:** 2017-01-31

**Authors:** Rahel Lenzlinger-Asprion, Niculina Keller, André Meichtry, Hannu Luomajoki

**Affiliations:** 1 Hauptstrasse 41, 8363 Bichelsee, Switzerland; 2 Tanne, 9573 Littenheid, Switzerland; 3 School of Health Professions, Institute of Physiotherapy, Zürich University of Applied Sciences, Technikumstrasse 71, CH-8401 Winterthur, Switzerland

**Keywords:** Movement control, Hip, Reliability, Assessment

## Abstract

**Background:**

Hip joint complaints are a problem associated with increasing age and impair the mobility of a large section of the elderly population. Reliable and valid tests are necessary for a thorough investigation of a joint. A fundamental function of the hip joint is movement control and a test of this function forms a part of the standard examination. Until now there have been few scientific studies which specifically investigate the reliability of measurement tests of movement control of the hip joint. The aim of this study was to examine the intratester and intertester reliability of the movement control tests of the hip joint which are in use in current clinical practice.

**Methods:**

Sixteen participants with hip joint complaints and 14 without hip joint impairment were recruited. All participants performed five active movement control tests for the hip joint and were video filmed whilst performing these tests. These films formed the basis for the evaluation and were assessed by two independent physiotherapists. For the intertester and intratester reliability calculations specially set weighted kappa values and the calculated percentages were used.

**Results:**

The intertester reliability of the five examined movement control tests of the hip joint showed good to almost perfect values (weighted kappa (wk) = 0.56–0.87). The intratester reliability of the more experienced evaluator A was better in regards to the less experienced evaluator B (average wk = 0.62 vs 0.38).

**Conclusion:**

The visual evaluation of movement control tests of the hip joint is especially reliable when carried out by an experienced evaluator. 4 out of 5 tests also showed good results for intertester reliability and support their use in clinical practice.

## Background

Hip joint complaints are a problem associated with increasing age and which impair the mobility of a large section of the elderly population. In older people the prevalence of hip pain is 20% [1] and for people with hip joint arthrosis the percentage rises to 27% [2, 3]. Different studies show that those people suffering from a hip joint dysfunction have a poorer quality of life in comparison to healthy people in the same age group [1, 4, 5]. The maintenance of a good quality of life is the main goal of physiotherapy. To be more precise, physiotherapists are in charge of the maintenance and/or improvement of the musculoskeletal system for regular everyday life activities [6]. In general, it is paramount to pinpoint the source of impairment or the supporting and favouring factors which lead to the problem. The practitioner relies upon a selection of evidence based tests [7] to make the relevant necessary clinical diagnosis. The aim is to use the most valid and reliable tests. The standard examination of the joint includes testing of the range of motion, muscular strength, muscle length and movement control.

Various methods for hip examination have already been tested for their intertester and intratester reliability. Different studies have examined various methods for measuring the joint’s range of motion. Depending on the study, the internal and external rotation of the hip joint was measured using electronic inclinometers, plurimeters or goniometers. The intratester reliability was found to be very high while the intertester reliability tended to be a little lower [8–11]. For flexion measurement, some studies also showed a good intertester reliability [10–12]. The calculated intertester and intratester reliability of the abduction and rotation strength measurement, postulated by Malliaras et al. [9] which was measured with an electronic dynamometer, had a range from ICC (intraclass correlation coefficient) 0.55–0.84 to respectively 0.40–0.73. These results are comparable with those of other studies [12, 13].

Functional tests have already been examined in various studies. Often the aim was to evaluate the general balance or postural control of elderly or mobility-impaired people to get a prediction of an existing risk of fall or as a protocol of a therapy [13, 14]. According to our research, there are very few studies to date which specifically examine the reliability of various movement control tests of the hip joint by means of visual evaluation. However, this is what physiotherapists do in their daily practice. Some studies evaluated the reliability of the One Leg Stand test. Here the focus was generally on the lumbar spine and the pelvis, but not on the movement control of the hip joint [15–17]. Furthermore, studies were found which examined the intertester and intratester reliability of the Single Leg Squat test. In these studies, however, the discussion focused mainly on the knee joint and movement patterns predeposed to cause knee problems [15, 18–20]. Only Monnier, Heuer, Norman and Ang [21] were found to have reported explicitly on the reliability testing of movement control tests in regards to the low back and the hip joint. In reality, however, only one test looked at movement control of the hip joint (single leg small knee bend + lunge-lean). The mentioned test over two rounds gave an intertester reliability of kappa (k) = 0.60 and 0.63 and an intratester reliability from k = 0.31 to 0.43. The study used a test-retest approach.

The aim of our study was to examine five different movement control tests of the hip joint which are currently in use in clinical practice and which, to date, have had no defined testing criteria with regard to their intertester and intratester reliability.

## Methods

### Study sample

Participants with and without hip problems (either clinical or radiographic signs of arthrosis) were included in the study.

Recruitment, which took place over 3 months, took place in the cantonal hospitals of Frauenfeld and Münsterlingen, Switzerland. Overall 16 participants with hip problems and 14 participants without hip joint impairment were included (Table 1). The age range of males and females was between 55–75 years.

**Table 1:** Demographic data

		Participants with hip joint complaints	Participants without hip joint complaints
Number of participants (m/f)		16 (6/10)	14 (8/6)
Working/Retired		4/12	8/6
Age (Years)			
mean (SD^a^, range)		67 (7, 55–74)	63 (5, 55–73)
Physical Active (min. 2×/week)		9 (56%)	13 (93%)
Cycling		5	3
Fitness		3	6
Gymnastics		0	2
Hiking		1	2
Long distance running/jogging		0	1
Pilates		1	0
Cross trainer		0	1
HOOS^b^ (0–100)			
mean (SD, range)		40 (18, 4–64)	–
Current Pain (NRS 0–10^c^)			
mean (SD, range)		2 (2, 0–6)	–
Diagnosis (Amount)		Duration of complaints (SD, range)	
THR^d^ after cox arthrosis	8	71 months (29, 36–120)	–
THR after femoral neck fracture	1	6 months	–
Hip dysplasia	1	6 months	–
Cox arthrosis	6	102 months (81, 36–240)	–

^a^standard deviation, ^b^hip osteoarthritis outcome score, ^c^numerical rating scale, ^d^total hip replacement

An inclusion criterion for participants with hip arthrosis was that they should be suffering from hip problems at the given time. At the time of recruitment, the participants were either in clinical care or shortly before a hip joint replacement operation or, due to their hip joint impairment, were out-patients under physiotherapy treatment.

The requirements of participants without hip problems were that they did not suffer from any hip impairment. The participants without hip impairment were out-patients under physiotherapy treatment due to problems of the thorax or upper limbs. Exclusion criteria were pain over the level of 5/10 on the Numeric Rating Scale (NRS), significant movement impairment in the lower extremities or back, current fractures, diseases which impact on active movements in standing positions (for example: dizziness).

All participants had to be able to understand the instructions in the German language. The aim of the study, as well as its background, was explained and all participants signed a written consent form prior to their participation.

Sample size analysis revealed, that with a similar distribution of correct and incorrect movement performances, 30 participants would be needed to verify a kappa value of 0.5 (power 80%) [22].

### Design

An intertester and intratester reliability study was performed according to the Declaration of Helsinki. The study was approved by the Ethics Committee of Canton Thurgau, Switzerland. Thirty participants performed five movement control tests of the hip and were filmed by video in a standardised manner from the ground to the shoulders. The video camera stood at a height midway between the knee and hip, centred on the patient at a distance away of 2–3 m.

Two physiotherapists, independent of the participants and each other, rated the videos twice as correct, almost correct or incorrect.

### Test protocol

In order to prevent a possible bias through recognition, to show the body section of the hip-pelvic-lumbar spine particularly well and to ensure the anonymity of the participants, all participants wore short black trousers during the test phase (women also wore a bra). The head was not filmed. The participants received a standardised oral instruction and were politely asked to follow these instructions as accurately as possible. If a participant could not perform the exercise according to the oral instructions, the movement was demonstrated and it was repeated a second time. Following this, the movement to be tested was filmed by video. The films were subsequently spliced into one single film. The order of the individual films was randomised. This video film was saved onto 2 DVDs and served as the basis for the evaluation.

The order of the performed tests was standardised: 1. Small Squat up to 30° (knee joint); 2. Squat up to 90° (hip joint); 3. One Leg Stand; 4. Small Single Leg Squat; 5. Step up.

### Description of the five tests for the movement control of the hip joint

#### Test 1: small squat up to 30° (the visual evaluation was frontal)

##### Standardised test instruction

**Fig. 1:**
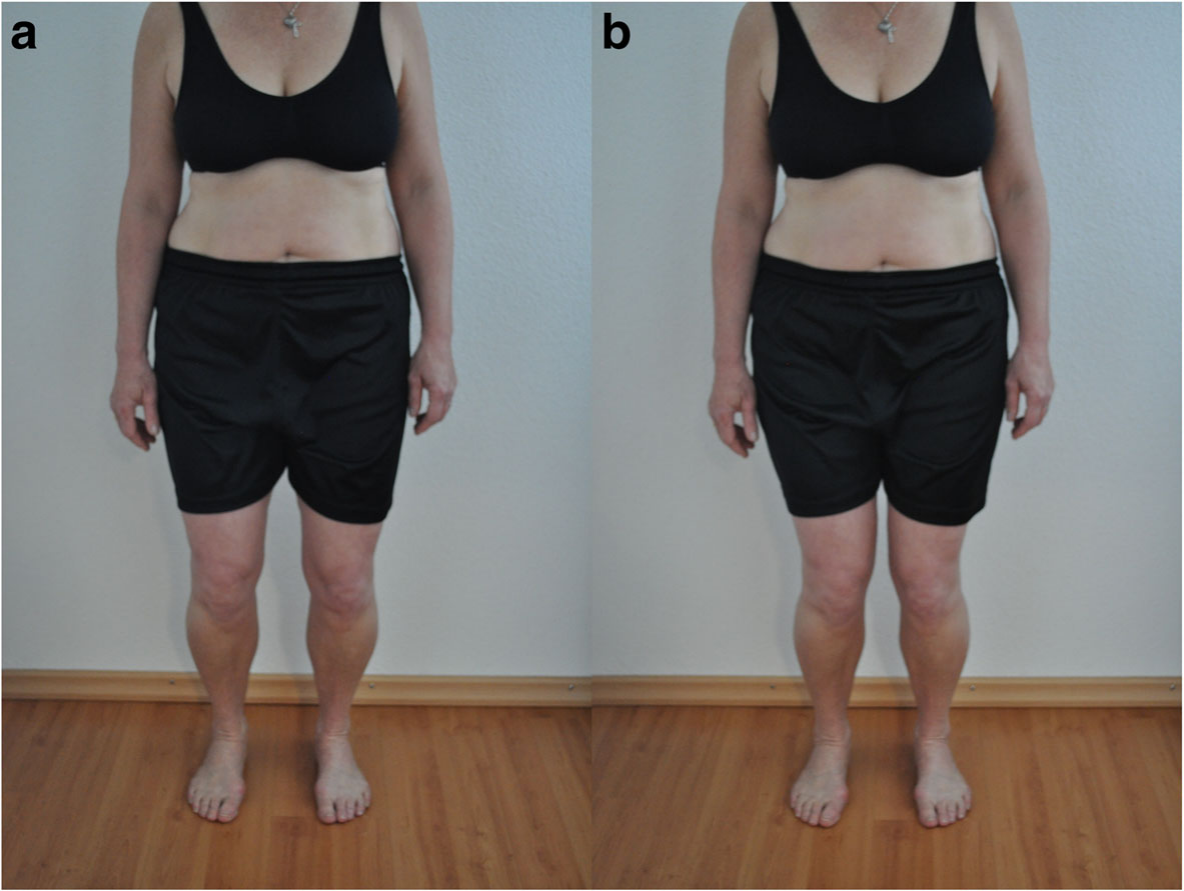
Test 1 «Small Squat up to 30°» (**a**) correct performance (2 points), (**b**) the second criteria is incorrect (1 point)

“First of all you take four stationary steps on the spot and remain standing on both feet afterwards (about hip-width apart). From this position, you will perform four small knee bends one after the other. The movement starts with the bending of the knee. The legs should stay in a vertically aligned. On the fourth repetition, please remain in the bended knee position for about 10 s (Table 2, Fig. 1).”

**Table 2:** Evaluation Criteria Small Squat up to 30° (knee joint)

1. Criteria	The performance of the movement should come initially from the knee joint and not from the hip joint. Continuous movement following the initial one may cause a small hip joint flection.
2. Criteria	The vertical axis of the length of the leg should remain straight. A genu varum or a valgum is not allowed to occur. The patella should point in the direction of the third metatarsale.

#### Test 2: squat up to 90° (the visual evaluation was frontal and afterwards from the side)

##### Standardised test instruction

**Fig. 2:**
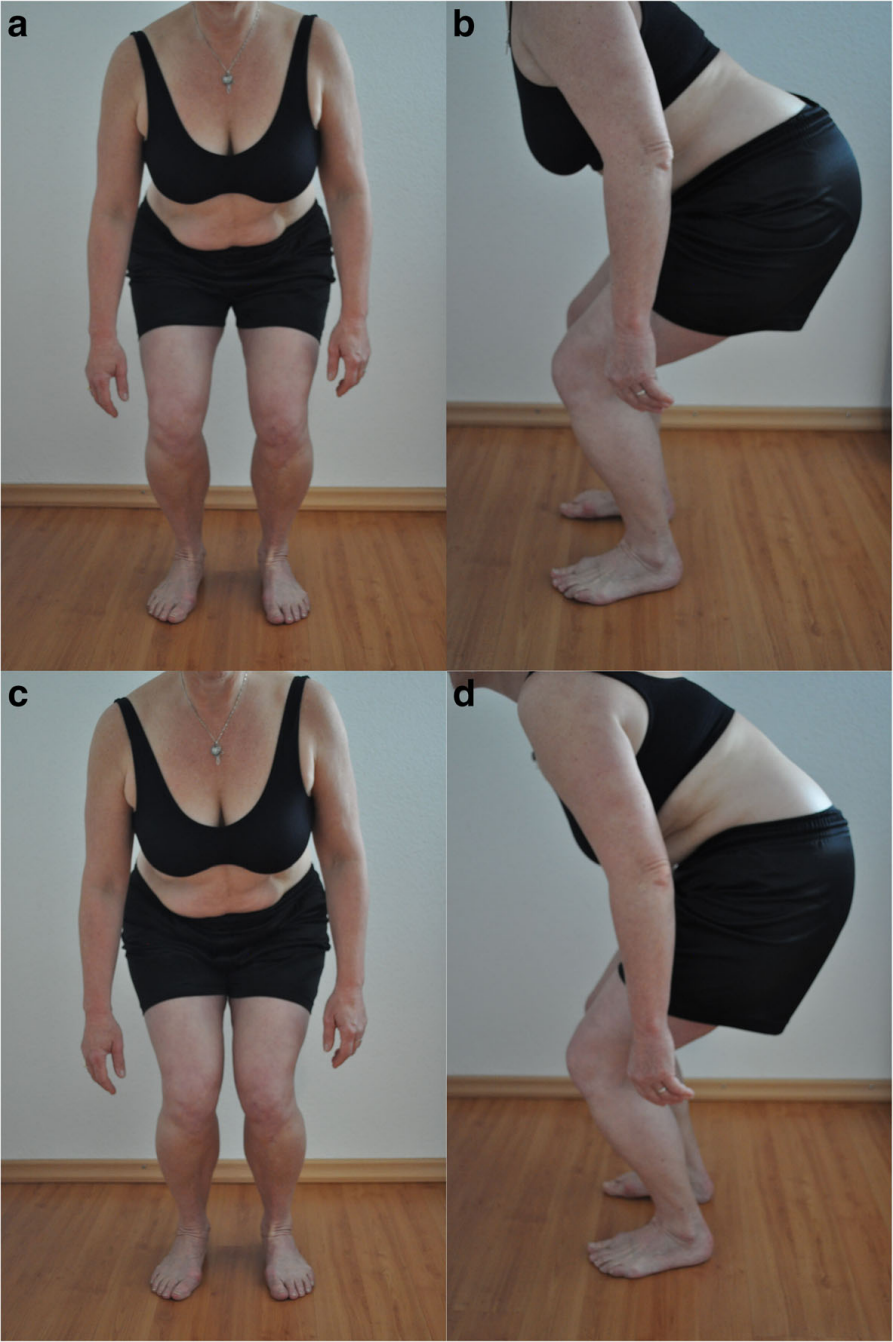
Test 2 «Squat up to 90°» (**a** and **b**) correct performance (2 points), (**c**) the second criteria is incorrect (1 point), (**d**) the second and third criteria are incorrect (0 points)

“First of all you take four stationary steps on the spot and remain standing on both feet afterwards (about hip-width apart). From this position, you will perform four small knee bends one after the other. The knees stay in a fixed position and then the movement begins with the backwards and downwards shifting of the pelvis. The fingertips move towards the knee cap. The position of the spine should not alter during the procedure. The legs should stay vertically aligned. On the fourth repetition, please remain in the squat position for about 10 s (Table 3, Fig. 2).”

**Table 3:** Evaluation Criteria Squat up to 90° (hip joint)

1. Criteria	The performance of the movement should come initially from the hip joint and not from the knee joint. The knees are allowed to move only slightly forwards. (maximum to the end of the toes)
2. Criteria	The vertical axis of the length of the leg should remain straight. A genu varum or a valgum should not occur. The patella should point in the direction of the third metatarsale.
3. Criteria	The spine should be kept in the neutral position.

#### Test 3: one leg stand (the visual evaluation was frontal)

##### Standardised test instruction

**Fig. 3:**
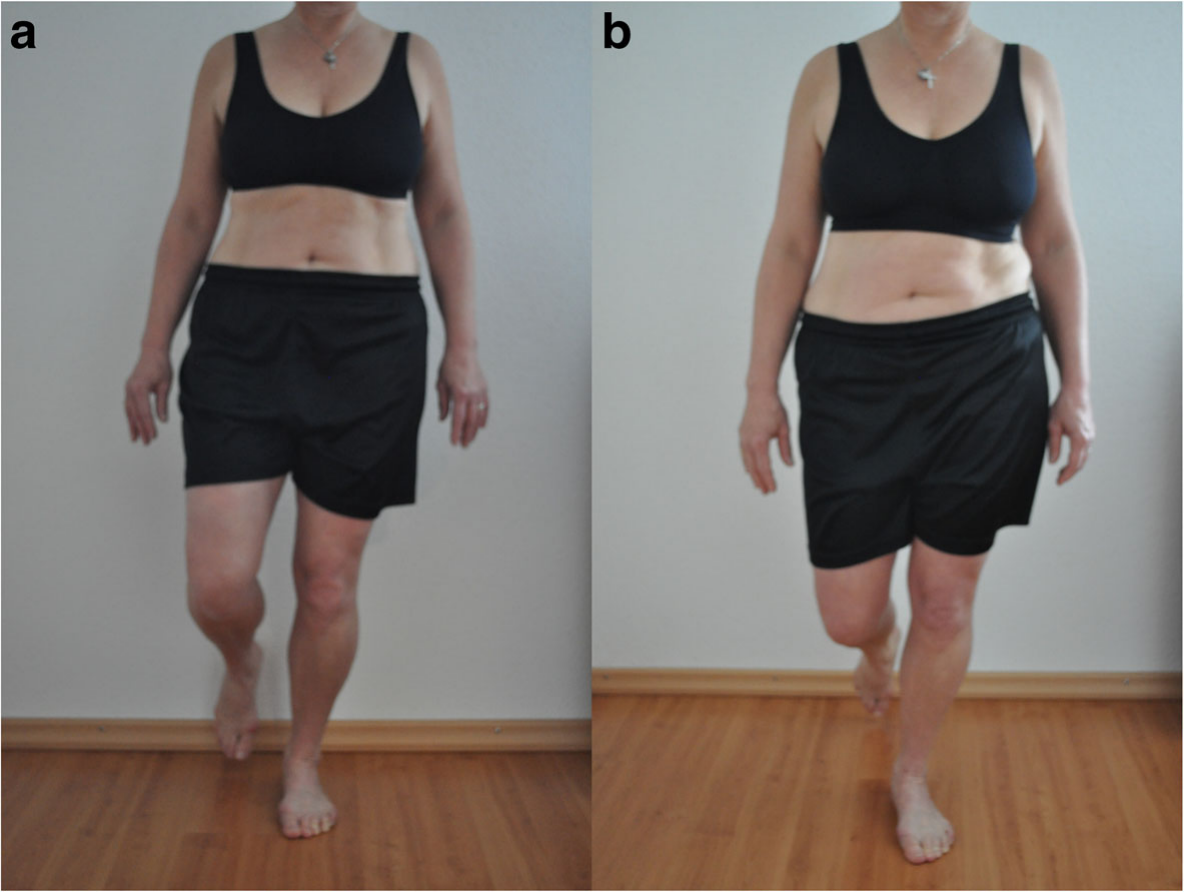
Test 3 «One Leg Stand» (**a**) correct performance (2 points), (**b**) the second criteria is incorrect (1 point)

“The aim is that you stand on one leg for about 10 s. The pelvis and the upper part of the body should not move and stay straight. The legs should also stay vertically aligned. Afterwards the same is repeated with the other leg (Table 4, Fig. 3).”

**Table 4:** Evaluation Criteria One Leg Stand

1. Criteria	The hip joint should remain stable in rotation, abduction and extension. Pelvis and the upper part of the body should not change from their initial position.
2. Criteria	The vertical axis of the length of the leg should remain straight. A genu varum or a valgum should not occur. The patella should point in the direction of the third metatarsale.
3. Criteria	If intermittent support is necessary with the hand against the wall or with the foot on the floor, the component is considered as incorrect. If additional support needed throughout the entire exercise, the component is valued as: >1 incorrect component

#### Test 4: small single leg squat (the visual evaluation was frontal)

##### Standardised test instruction

**Fig. 4:**
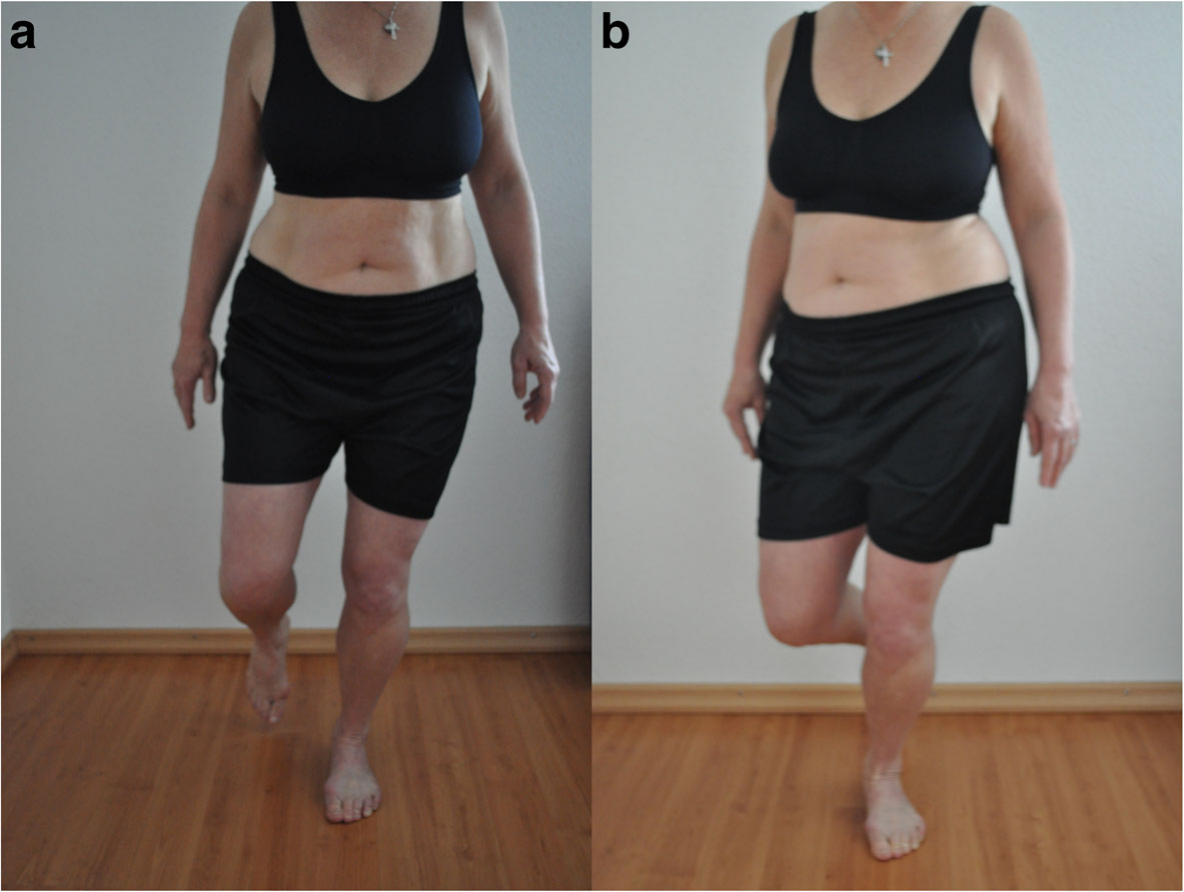
Test 4 «Single Leg Squat up to 30°» (**a**) correct performance (2 points), (**b**) the second and third criteria are incorrect (1 point)

“First of all you take the position of the One Leg Stand as previously performed. Starting from this position, as in the very first test, you will perform four small knee bends one after the other. The movement starts with the bending of the knees. The pelvis and the upper part of the body should not move and stay straight. The legs should also stay vertically aligned. On the fourth repetition, please stay in the squat position for about 10 s. When feeling unstable, a one-off support with the foot on the floor or the hand against the wall is allowed (Table 5, Fig. 4).”

**Table 5:** Evaluation Criteria of the Small Single Leg Squat

1. Criteria	The performance of the movement should come initially from the knee joint and not from the hip joint. Continuous movement following the initial one may cause a small hip joint flection.
2. Criteria	The hip joint should remain stable in rotation, abduction and extension. Pelvis and the upper part of the body should not change from their initial position.
3. Criteria	The vertical axis of the length of the leg should remain straight. A genu varum or a valgum should not occur. The patella should point in the direction of the third metatarsale.
4. Criteria	If intermittent support is necessary with the hand against the wall or with the foot on the floor, the component is considered as incorrect. If additional support needed throughout the entire exercise, the component is valued as: > 2 incorrect criteria

#### Test 5: step up (the visual evaluation was frontal, step height 15 cm)

##### Standardised test instruction

**Fig. 5:**
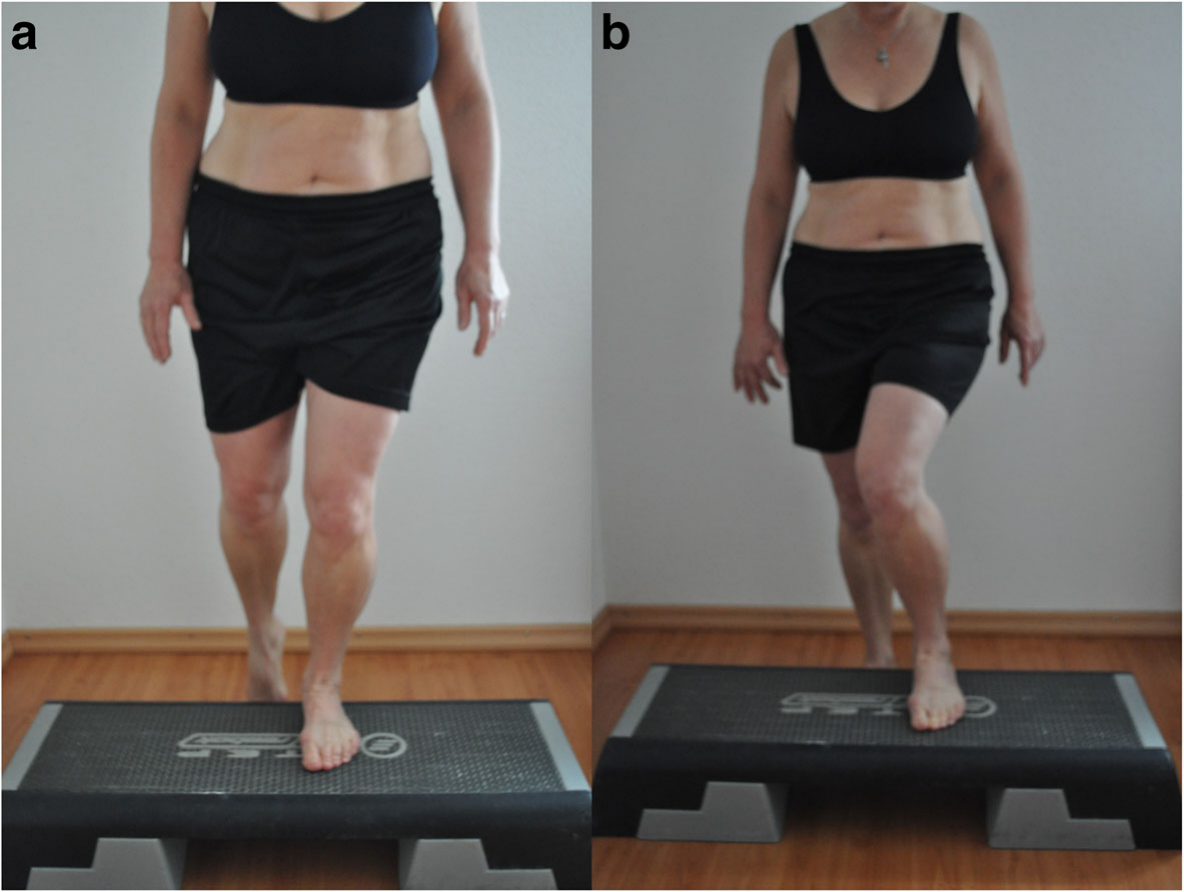
Test 5 «Step up» (**a**) correct performance (2 points), (**b**) the second criteria is incorrect (1 point)

“You are standing in front of an aerobic step and, using the same leg, you should step up and down four times. Afterwards the same is performed with the other leg. (For example, right leg goes up first and right leg goes down first). The pelvis and the upper body should not move and stay straight. The legs should also stay vertically aligned (Table 6, Fig. 5).”

**Table 6:** Evaluation Criteria of the Step up

1. Criteria	The hip joint should remain stable in rotation, abduction and extension. Pelvis and the upper part of the body should not change from their initial position.
2. Criteria	The vertical axis of the length of the leg should remain straight. A genu varum or a valgum should not occur. The patella should point in the direction of the third metatarsale.

As the examination relies purely on inspection, it can be difficult to see the faulty movements in a dynamic movement. Therefore the alignment was also evaluated through static posture and this is why the participants had to stop and hold the position after the last repetition.

When the majority of the movements are performed correctly, the components will be evaluated as correct. When the majority of the movements are performed incorrectly, the components will be evaluated as incorrect. In the case, where only half the movements are performed correctly, a subjective evaluation will be made based on the magnitude of the deviation and the probability of a randomly correct execution.

### Rating of test performance

The evaluators were blinded to each other. One evaluator has been qualified for over 20 years and has successfully performed several courses in manual therapy and functional kinetics. The second evaluator had been qualified for 4 years and has also participated successfully in courses in manual therapy. The evaluators were trained on the evaluation criteria prior to the actual evaluation in a workshop. They had to evaluate seven examples of each test movement. At the end of this workshop there was sufficient time to discuss the results. The criteria of evaluation (Tables 2, 3, 4, 5 and 6) were explained precisely and discussed with the help of filmed examples. A DVD, together with the first evaluation form, was given to each of the evaluators at the end of the workshop. For the analysis of intratester reliability they performed two rounds of actual evaluation. After the first round the evaluators had to wait 7 days before they were allowed to perform the second round of evaluation. The second form was given to them upon handing in of the first evaluation form. The evaluators were allowed to watch the films several times, but they were not allowed to slow down the film. The evaluators were blinded to the participants as well as to their medical diagnosis.

The evaluation took place using a 3-point Likert scale (Table 7): 2 points = correct; 1 point = almost correct; zero points = incorrect/false. The evaluation of the One Leg Stand tests was carried out using the impaired side of participants with hip problems, whilst the side to be tested for participants without hip problems was chosen randomly. The assessment of the evaluation forms from the two independent evaluators was done by RL who was uninformed with regards to both evaluators A and B.

**Table 7:** Rating of Tests

Rating	Test 1–3 and 5	Test 4
2 = correct	all criteria are correct	all criteria are correct
1 = almost correct	1 criteria is incorrect	≤2 criteria are incorrect
0 = incorrect	>1 criteria are incorrect	>2 criteria are incorrect

### Statistical analysis

The statistical analysis was conducted using the software package R. For intertester and intratester reliability the weighted kappa coefficient (wk) had a 95% confidence interval (CI) and the percentage of agreement was calculated for each test.

According to Landis et al. [23], wk > 0.80 was defined as almost perfect, 0.60–0.80 as substantial, 0.40–0.60 as good, 0.20–0.40 as fair and <0.20 as poor.

For a sufficient level of reliability, tests should reach at least a kappa of >0.40 and a lower bound of confidence interval of >0.2.

## Results

Table 8 shows the attained values for intertester reliability of the weighted kappa, the 95% CI and the percentage of agreement with each test from the first rating. Three tests out of five had a substantial (wk = 0.66) and two tests showed a good intertester reliability (wk = 0.52). The lower bound of 95% CI was only found to be under 0.20 in test 1. The percentage agreement was from 62 to 73%.

**Table 8:** Intertester and Intratester Reliability

	Test 1	Test 2	Test 3	Test 4	Test 5
Intertester A vs B^a^					
Matches (%)	22/30 (73%)	21/30 (70%)	21/30 (70%)	21/30 (70%)	18/29 (62%)
weighted kappa (95% CI^b^)	0.52 (0.17–0.86)	0.71 (0.53–0.89)	0.68 (0.44–0.92)	0.66 (0.46–0.86)	0.52 (0.21–0.81)
Intratester A					
Matches (%)	25/30 (83%)	25/30 (83%)	25/29 (86%)	23/30 (77%)	18/29 (62%)
weighted kappa (95% CI^a^)	0.76 (0.62–0.91)	0.80 (0.63–0.96)	0.87 (0.75–0.99)	0.78 (0.64–0.93)	0.56 (0.32–0.80)
Intratester B					
Matches (%)	24/30 (80%)	16/30 (53%)	16/30 (53%)	21/30 (70%)	16/29 (55%)
weighted kappa (95% CI)	0.55 (0.21–0.88)	0.35 (0.07–0.63)	0.55 (0.33–0.76)	0.61 (0.40–0.82)	0.55 (0.34–0.76)

^a^1. Set of Evaluation, ^b^95% confidence interval

Table 8 shows the attained values for intratester reliability (wk, CI, agreement).

For test 3 rater A showed an almost perfect reliability (wk = 0.87), for tests 1, 2 and 4 a substantial reliability (wk = 0.76) and for test 5 a good reliability (wk = 0.56).

Rater B had a substantial reliability (wk = 0.61) for test 4. The other tests were rated as good to fair. Only rater B showed a value for one test under the lower bound of 0.20 of 95% CI (test 2).

Average HOOS score was 40 points out of 100 (moderate disability).

## Discussion

The aim of this study was to investigate the intertester and intratester reliability of five movement control tests of the hip for patients with arthrosis. The tests demonstrated higher intratester reliability (wk = 0.52–0.71). The more experienced rater had better values in the intratester reliability.

The good intertester reliability was thought to be due to the workshop where both testers were trained onto which much attention was placed beforehand. The difference of the intratester reliability of the evaluators may be due to the difference in years of working experience: 20 years compared to 4 years. This hypothesis is discussed controversially in other studies due to varying results [18, 20, 24].

Although the tests are designed for patients with hip problems, it is important to evaluate the whole movements and also the neighboring segments of the body. So, for example a weakness of the Gluteal muscles presents as a lateral deviation of the trunk (“Duchenne sign”). Or the weakness of the Quadriceps, especially of the medial part, shows as an adduction of the knee.

Some of the tests used in this study were previously tested for intertester and intratester reliability and reached moderately good to almost perfect values [15–19, 21]. Even though the mentioned studies examined different participant groups, for example patients with low back pain, marines on active duty or a population with a mean age of 25 years, the results can be compared due to the similarity in method. The Single Leg Squat has been the most examined test. Interestingly, the intertester reliability was found to be the best when the physiotherapist had a lot of experience and when the evaluator was trained beforehand in previous studies [18–20]. In the study of Harris-Hayes et al. (2014), 2 of 3 evaluators had an average of 18 years work experience and had created the tests and their criteria themselves. When evaluating the knee alignments (angle doesn’t change/>10°, change to medial/>10° change to lateral) they reached an intertester reliability of wk = 0.9. Together with the third evaluator who had no clinical experience but who was also trained, an intertester reliability of wk = 0.75 was achieved. Similar tendencies were also noted in studies in which movement of the lower back was evaluated visually according to predefined criteria [24, 25].

The criteria of evaluation in this study could be considered most similar to study of Poulsen et al. [15] and Crossley et al. [20], in which the Single Leg Squat included the torso, the pelvis, the hip and the knee joint in the evaluation (results in Table 9). In the study of Tidstrand et al. [16], the position of the lower back and the pelvis were evaluated using the One Leg Stand. The evaluators with 5 years of experience underwent a similar training as the evaluators in this study. They reached an average intertester reliability of k = 0.94. Three of 19 tests were regarded as positive. The unequal distribution of the test results could have influenced the study results for the better. To compare the results, only the intratester reliability of the Small Single Leg Squat could be found. In the current study, the more experienced evaluator reached much better results (on average wk = 0.75 vs 0.52). There are studies supporting these results, indicating better intratester reliability for evaluators with more experience [20], but there are also studies showing contrary findings [15, 18] (Table 9).

**Table 9:** Comparison with other studies which evaluated reliability

Study	Intertester Reliability		Intratester Reliability	
One Leg Stand	Much Experience	Little Experience	Much Experience	Little Experience
This study		wk = 0.68	wk = 0.87	wk = 0.55
Roussel et al., 2007 [17] (patients with LBP, 18–65 years)		wk = 0.79		
Tidstrand & Horneij, 2009 [16] (patients with LBP or shoulder pain, 18–65 years)	k = 0.94			
Small Single Leg Squat				
This study		wk = 0.66	wk = 0.78	wk = 0.61
Ageberg et al., 2010 [19] (healthy and young participants)	k = 0.92^d^			
Crossley et al., 2011 [20] (healthy and young participants)	k = 0.7–0.8	k = 0.6	k = 0.8, 0.692	k = 0.613
Harris-Hayes et al., 2014 [18] (Graduate/undergraduate students without injury)	wk = 0.9	wk = 0.75^a^	wk = 0.9, 0.8	wk = 0.84^a^
Poulsen & James, 2011 [15]^c^ (healthy lower extremity, 18–60 years)		wk = 0.68^b^		wk = 0.94–0.38^b^

*k* kappa, *wk* weighted kappa, *LBP* Low Back Pain, ^a^no clinical experience, ^b^physiotherapist student, ^c^only evaluator without schooling, ^d^no information given, much experience is regarded after 5 years of work experience

### Strengths and limitations of this study

The video film recordings were an ideal method for the analysis in this study since both therapists viewed exactly the same material from the same perspective. Moreover, a maximum of blinding regarding the group association of the participants was ensured. Neither habitual nor pain-related movements could be seen nor sounds which were made before, during or after the test could be heard, which might have distracted the evaluators. Nevertheless, it must be noted that assessment by video is a deviation from clinical practice and that there is a difference between video analysis and analysis in clinical practice. Another advantageous aspect was that the tests were uncomplicated and fast to perform. The only supplementary equipment required was an aerobic step.

The results of this study should be viewed with regard to various limitations. It is possible that it was a challenge for the evaluators to maintain the same level of concentration for the entire duration of the evaluation (about 2 h). A decline in motivation and concentration could have had an impact on the evaluation. The realization of the movements could have been standardised even more precisely. Similar studies worked, for example, with a metronome [19] or with an electronic goniometer [15] in order to standardize the speed and the depth of movement. In parts, even the position of the non-supporting leg was standardised [16, 18].

### Further research

In further studies the test-retest reliability should be examined so that the results can be even more usefully applied in clinical practice. Furthermore, studies describing validity must follow. For this, the sample size needs to be larger. Moreover, a more homogenous group with regard to the complaints of the participants should be considered for study.

## Conclusion

This study shows a good to substantial intertester reliability. We propose the use of the Squat, One Leg Stand, Small Single Leg Squat and Step up tests. The Small Squat test resulted in a bad 95% confidence interval. These tests could be used to measure treatment progress and outcome in clinical practice. A general recommendation is that the tests be performed by the same experienced physiotherapist because the intratester reliability was better than the intertester reliability.

## Abbreviations

CI: Confidence interval
HOOS: Hip osteoarthritis outcome score
ICC: Interclass correlation coefficient
k: Kappa
NRS: Numeric rating scale
SD: Standard deviation
THR: Total hip replacement
wk: Weighted kappa
